# Supplementary figures and images for: Reprograming gene expression in ‘hibernating’ C. elegans involves the IRE-1/XBP-1 pathway
Source: eLife. 2025 May 6;13:RP101186. doi: 10.7554/eLife.101186 (PMC12055002; doi:10.7554/eLife.101186)

Puromycin low exposure

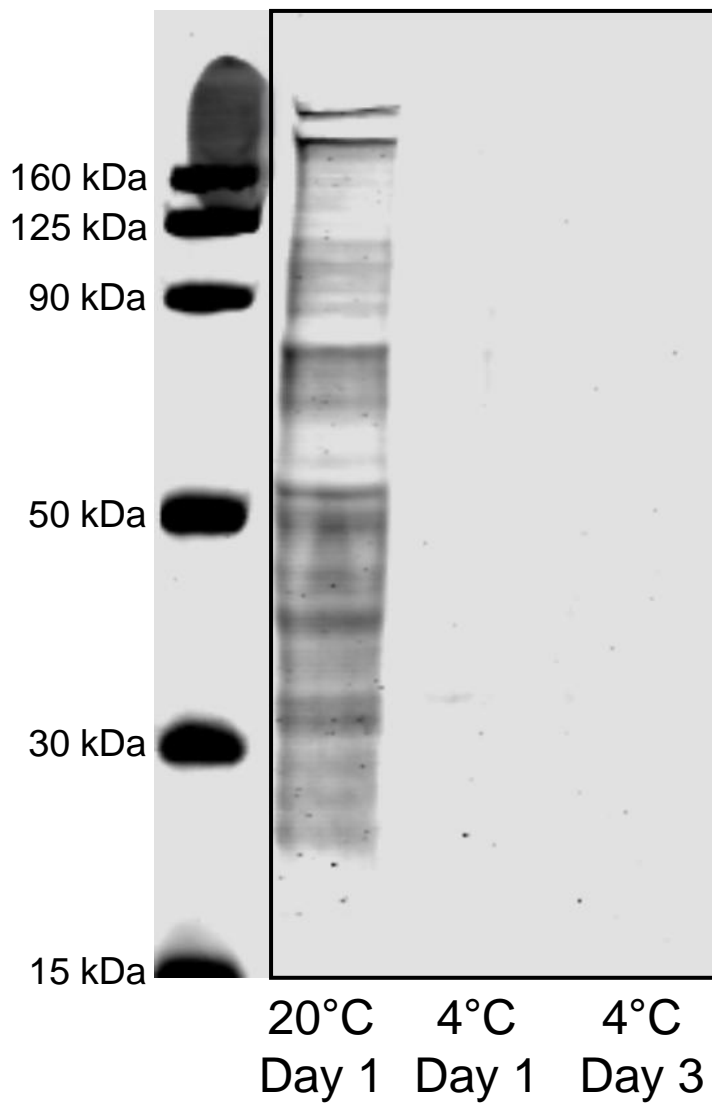

Puromycin high exposure

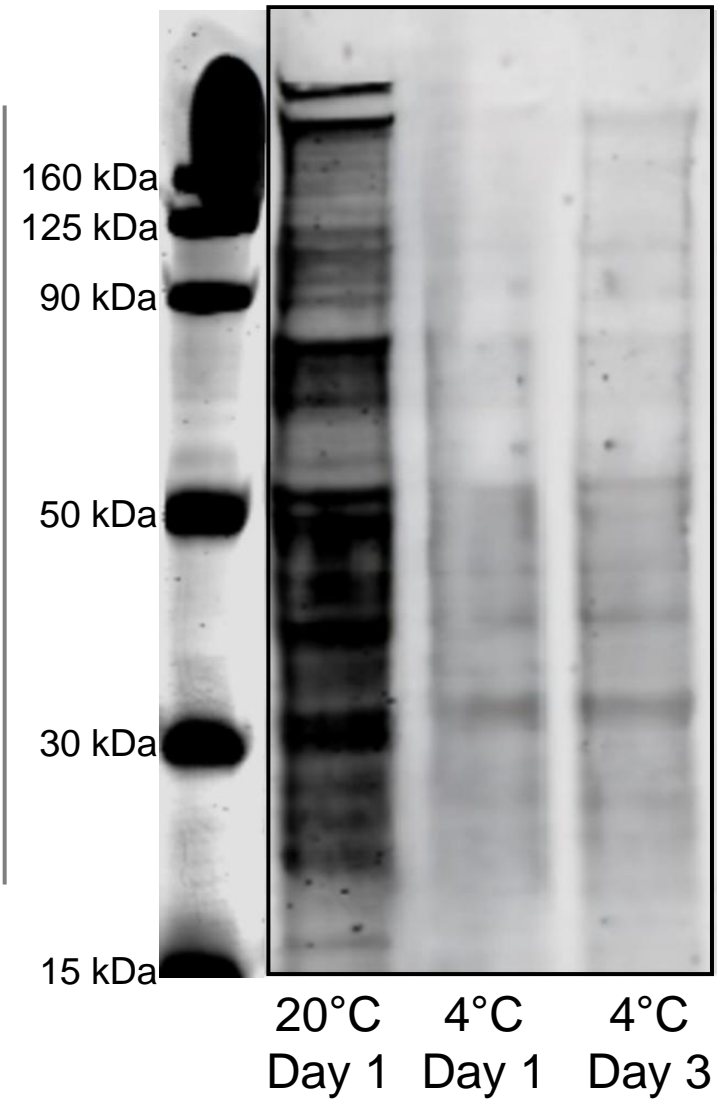

Actin

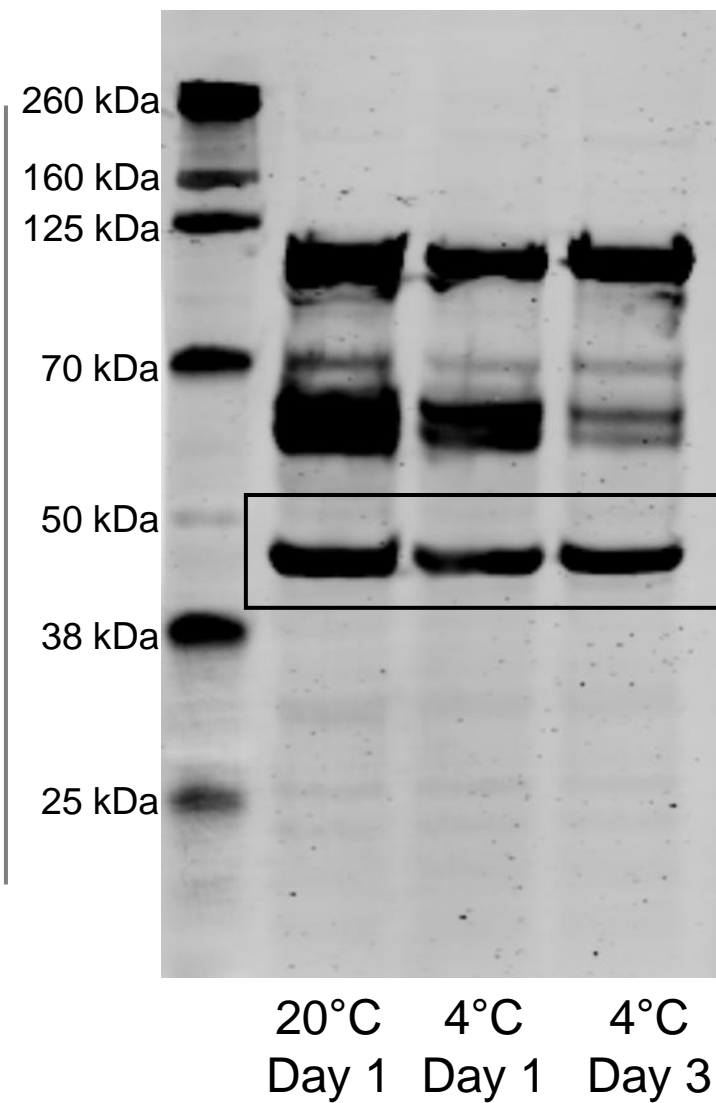

Supplement: Figure 1—figure supplement 1—source data 1. [file elife-101186-fig1-figsupp1-data1.zip › Figure 1 – figure supplement 1-source data 1/Source data 1.pdf]

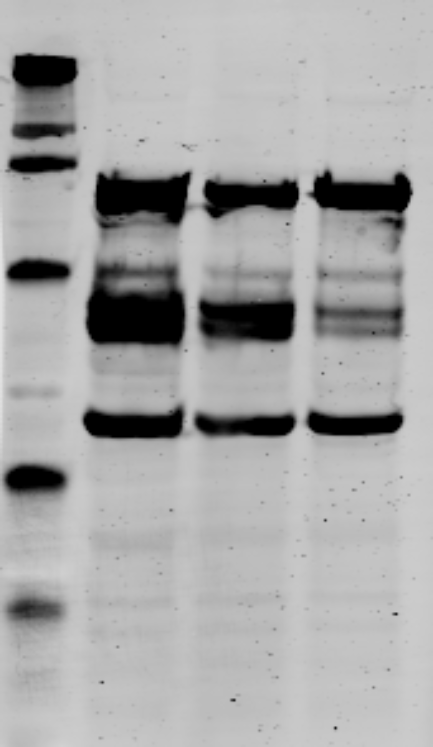

Supplement: Figure 1—figure supplement 1—source data 2. [file elife-101186-fig1-figsupp1-data2.zip › Figure 1 – figure supplement 1-source data 2/Actin.tif]

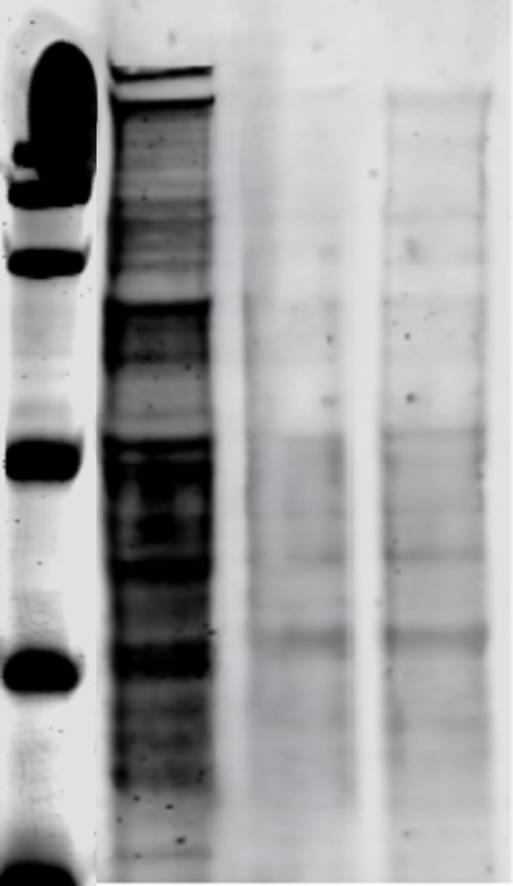

Supplement: Figure 1—figure supplement 1—source data 2. [file elife-101186-fig1-figsupp1-data2.zip › Figure 1 – figure supplement 1-source data 2/Puromycin high exposure.tif]

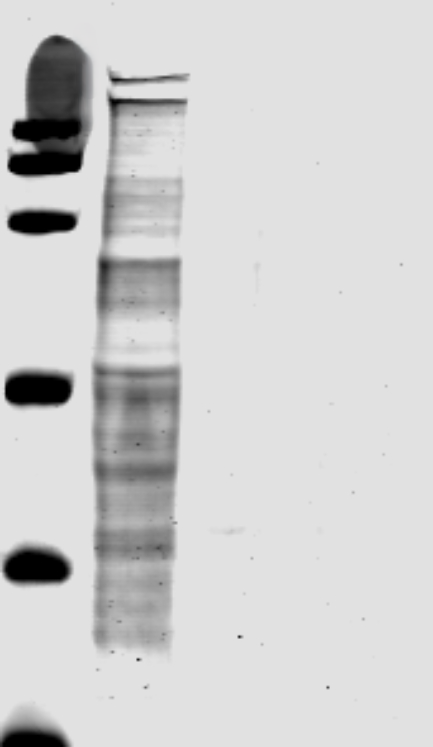

Supplement: Figure 1—figure supplement 1—source data 2. [file elife-101186-fig1-figsupp1-data2.zip › Figure 1 – figure supplement 1-source data 2/Puromycin low exposure.tif]
