# Supplementary material for: Reprograming gene expression in ‘hibernating’ C. elegans involves the IRE-1/XBP-1 pathway
Source: eLife. 2025 May 6;13:RP101186. doi: 10.7554/eLife.101186 (PMC12055002; doi:10.7554/eLife.101186)
Supplement: Supplementary file 6. [file elife-101186-supp6.docx]

**Supplementary file 6A: The *C. elegans* strains used in this study.**

| **Genotype** | **Source** | **Identifier** |
| --- | --- | --- |
| Wild type | CGC | N2 |
| P*lips-11::GFP::unc-54* 3’UTR | This study | RAF5141 |
| zcIs4[P*hsp-4::GFP*] | CGC | SJ4005 |
| zcIs13 [*hsp-6p::GFP* + *lin-15*(+)] | CGC | SJ4100 |
| zcIs9 [*hsp-60::GFP* + *lin-15*(+)] | CGC | SJ4058 |
| rmsIs8[P*xbp-1::xbp-1::GFP*] | Özbey et al, 2020 (PMID: 33232669) | RCT21 |
| hhIs113[P*nhx-2::cpl-1^W32AY35A^::YFP ;* P*myo-2::mCherry*] | Efstathiou et al, 2022 (PMID: 36471127) | PP1386 |
| *ire-1(ok799)* | CGC | RB925 |
| mtEx63[*numr-1p::numr-1::GFP + rol-6(su1006)*] | CGC | JF88 |
| wgIs563[*cebp-1::TY1::EGFP::3xFLAG + unc-119(+)*] | CGC | OP563 |
| st12254[*hsf-1::TY1::EGFP::3xFLAG*] | CGC | RW12254 |

**Supplementary file 6B: Oligos used in this study.**

| **Name** | **Sequence 5’ 🡪 3’** | **Purpose** |
| --- | --- | --- |
| P*lips-11* FWD | GGGGACAACTTTGTATAGAAAAGTTGTCaatgggcggagcctattatt | Gateway cloning |
| P*lips-11* REV | GGGGACTGCTTTTTTGTACAAACTTGCtcgggatctggaaaaaaa |  |
| GFP FWD | GGGGACAAGTTTGTACAAAAAAGCAGGCTTCACCATGAGTAAAGGAGAAGAACT |  |
| GFP REV | GGGGACCACTTTGTACAAGAAAGCTGGGTCTATTTGTATAGTTCATCCATGCCATGT |  |
| *act-1* FWD | TTGCCCCATCAACCATGAAGA | RT-qPCR |
| *act-1* REV | TGTGCAAGTTGACGAAGTTGTG |  |
| *lips-11* FWD | ACCATTGTCCAAGCGGAGTT |  |
| *lips-11* REV | AGAGGGACTCTTCTTGTTTGAGT |  |
| *hsp-4* FWD | GGCAAACGCGTACTGTGATG |  |
| *hsp-4* REV | ACGCAACGTATGATGGAGTGATT |  |
| *dnj-27* FWD | CGTTTTACGACTACCCGAAC |  |
| *dnj-27* REV | CCATACCACTGTCCTGGACTC |  |
| *srp-7* FWD | CTCAGATCTCGTCGCTGTTC |  |
| *srp-7* REV | GATTTTCTCCATGCCTCCAGTG |  |
| *C36B7.6* FWD | CTTCCAGTCCCTCATTGTCTTC |  |
| *C36B7.6* REV | GAACCAATCCGATTCTTGGCG |  |
| *cht-1* FWD | GGCTCAATACAGACAAGGACG |  |
| *cht-1* REV | CGCTGACTACACCGTTCGAG |  |
| *ZC168.2* FWD | CATGTCCGGTGTTAGCTGATG |  |
| *ZC168.2* REV | CAGAGCCGACTGCTTCACAAC |  |
| *cbp-3* FWD | GTCATTTCGCTCATTGTGCATC |  |
| *cbp-3* REV | CATTGCCCGATGTGATCAGTC |  |
| *R02D3.8* FWD | CAGTAGCCCATTACTCATCATTC |  |
| *R02D3.8* REV | TGAGCCAGCACTTTGATTATC |  |
